# Secondary Small Interfering RNA-Based Silencing Tools in Plants: An Update

**DOI:** 10.3389/fpls.2019.00687

**Published:** 2019-05-28

**Authors:** Alberto Carbonell

**Affiliations:** Instituto de Biología Molecular y Celular de Plantas, Consejo Superior de Investigaciones Científicas-Universitat Politècnica de València, Valencia, Spain

**Keywords:** RNA silencing, secondary siRNA, tasiRNA, atasiRNA, syn-tasiRNA, MIGS

## Plant Secondary Small Interfering RNAs

In plants, RNA silencing regulates key biological processes such as development, response to stress, genome integrity, and antiviral resistance. RNA silencing functions through diverse classes of small RNAs (sRNAs) that associate with ARGONAUTE (AGO) proteins to repress highly sequence-complementary target transcripts (Baulcombe, 2004).

Small interfering RNAs (siRNAs) are a class of sRNAs arising from double-stranded RNA (dsRNA) precursors. Secondary siRNAs are those siRNAs whose dsRNA precursor synthesis is triggered by an upstream sRNA-guided transcript cleavage event followed by RNA-dependent RNA polymerase (RDR) activity (for a recent review see de Felippes, 2019). Many secondary siRNAs are produced in 21-nucleotide (nt) register with the sRNA-guided cleavage site by successive Dicer-Like (DCL) processing and are therefore called phased secondary siRNAs (phasiRNAs). In contrast, only a subset of secondary siRNAs act *in trans* to repress one or more targets distinct from their locus of origin. These siRNAs are called trans-acting siRNAs (tasiRNAs), most of which are also phased (Axtell, 2013).

## Classes of Secondary siRNA-based Silencing Tools

While secondary siRNAs may be ultimately generated in classic RNA interference (RNAi) approaches such as virus induced gene silencing (VIGS) or hairpin-based silencing after the initial targeting of transgene-derived (primary) siRNAs (Ossowski et al., 2008), only two classes of silencing tools operate primarily through the action of secondary siRNAs. These are (i) artificial or synthetic tasiRNAs (atasiRNAs and syn-tasiRNAs, respectively, both terms are accepted), and (ii) miRNA-induced gene silencing (MIGS). Both classes of secondary siRNA-based tools have been extensively used in plants to induce selective gene silencing in basic gene function studies or to improve agronomic traits.

**Figure 1:**
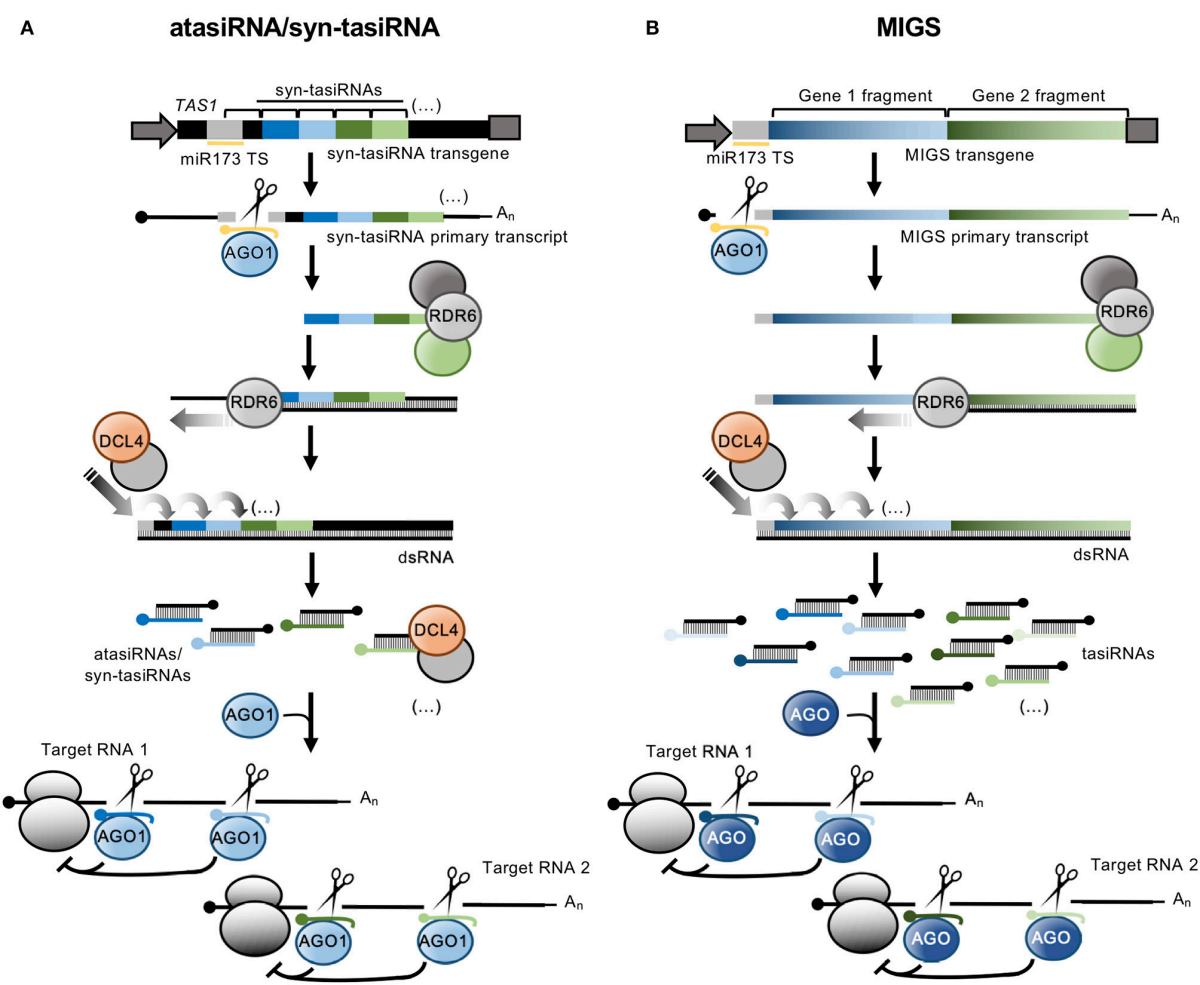
Secondary siRNA-based silencing tools in plants. **(A)**. The atasiRNA/syn-tasiRNA pathway. *TAS1* precursor sequence is in black, with trigger miR173 target site (TS) in light gray. AtasiRNA/syn-tasiRNA sequences targeting RNA 1 are shown in dark and light blue; atasiRNA/syn-tasiRNA sequences targeting RNA 2 are shown in dark and light green; miR173 sequence is shown in yellow. Promoter and terminator sequences are shown with a dark gray arrow and box, respectively. Participating proteins are represented with colored ovals. **(B)** The MIGS pathway. Sequences corresponding to gene fragments 1 and 2 are in diverse blue and green tonalities, respectively. Other details are as in **(A)**.

atasiRNA/syn-tasiRNAs are expressed from transgenes including engineered *TAS* precursors in which a region corresponding to various endogenous tasiRNAs is substituted by a fragment containing multiple atasiRNA/syn-tasiRNA sequences (Figure 1A). In *Arabidopsis thaliana* (Arabidopsis), modified *TAS* transcripts are cleaved by a specific microRNA (miRNA)/AGO complex (e.g., miR173/AGO1 and miR390/AGO7 cleave *TAS1*- and *TAS3*-based precursors, respectively), and one of the cleavage products is converted by RDR6 to dsRNA, which is processed by DCL4 into phased tasiRNA duplexes in 21-nt register with the miRNA cleavage site. atasiRNA/syn-tasiRNA guide strands, typically designed to contain an AGO1-preferred 5' U, are incorporated into AGO1 to direct silencing of one or multiple transcripts at one or multiple sites (Figure 1A). Importantly, the multiplexing of several atasiRNAs/syn-tasiRNAs in a single construct allows for the efficient and simultaneous multitargeting of various sequence-related or unrelated genes. Moreover, as for artificial miRNAs (amiRNAs), atasiRNAs/syn-tasiRNAs can be computationally designed with user-friendly web tools such as P-SAMS (http://p-sams.carringtonlab.org/) (Fahlgren et al., 2016) to be highly specific and prevent the so-called off-target effects characteristic of other RNAi approaches. For instance, P-SAMS designs artificial sRNAs that contain (i) an AGO1-preferred 5' U, (ii) a C in position 19 to generate a star strand with an AGO1 non-preferred 5' G thus avoiding competition for AGO1 loading, and (iii) an intentional mismatch with the target transcript at position 21 to limit possible transitivity effects (Carbonell, 2017). Initially, atasiRNAs/syn-tasiRNAs were used to efficiently repress one or multiple endogenous genes in gene function studies in Arabidopsis (de La Luz Gutierrez-Nava et al., 2008; Montgomery et al., 2008a,b; Carbonell et al., 2014) (Table 1). More recently, atasiRNAs/syn-tasiRNAs have emerged as an effective approach to induce resistance against viruses and viroids in several plant species (Chen et al., 2016; Carbonell and Daròs, 2017; Carbonell et al., 2019) (Table 1), and, more broadly, as a promising tool for plant biology study and crop improvement (for a review see Zhang, 2014).

**Table 1:** Uses of secondary siRNA-based tools in plants.

**Secondary siRNA tool**	**Precursor**	**miRNA trigger**	**Plant species**	**Target(s)^a^**	**References**
atasiRNA/syn-tasiRNA	*TAS1a*	miR173	*Arabidopsis thaliana*	*SUL*	Felippes and Weigel, 2009 Baykal et al., 2016
	*TAS1c*			*CPC, ETC2, FT, TRY*	Carbonell et al., 2014
				*FAD2*	de La Luz Gutierrez-Nava et al., 2008
				*PDS*	Montgomery et al., 2008b
			*Nicotiana benthamiana*	PSTVd	Carbonell and Daròs, 2017
				TSWV	Carbonell et al., 2019
	*TAS3*	miR390	*Arabidopsis thaliana*	*PDS*	Montgomery et al., 2008a
				CMV, TuMV	Chen et al., 2016
MIGS	–	miR173	*Arabidopsis thaliana*	*AG, ELF3, FT, GFP, LFY*	de Felippes et al., 2012
				*CH42*	Felippes and Weigel, 2009
				*GFP*	Martínez et al., 2016
				*miP1a, miP1b*	Graeff et al., 2016
				*PDS*	Sarrion-Perdigones et al., 2013
				*PGDH1*	Benstein et al., 2013
				*PSAT1*	Wulfert and Krueger, 2018
			*Capsella rubella*	*RPP5*	Sicard et al., 2015
			*Medicago truncatula*	*CEP1*	Imin et al., 2013
			*Oryza sativa*	*GBSS, LAZY1, PDS, ROC5*	Zheng et al., 2018
			*Petunia hybrida*	*CHS, PDS*	Han et al., 2015
		miR390	*Arabidopsis thaliana*	*CH42*	Felippes and Weigel, 2009
			*Nicotiana tabacum Solanum lycopersicum*	ToLCNDV, ToLCGV	Singh et al., 2015
		miR1514a.2	*Glycine max*	*NFR1α, P450 CYP51G1*	Jacobs et al., 2016
	*TAS1c*	miR173	*Nicotiana benthamiana*	PPV	Zhao et al., 2015

a *AG, AGAMOUS; CEP1, C-TERMINALLY ENCODED PEPTIDE 1; CH42, CHLORINA 42; CHS, CHALCONE SYNTHASE; CMV, Cucumber mosaic virus; CPC, CAPRICE; ELF3, EARLY FLOWERING 3; ETC2, ENHANCER OF TRIPTYCHON AND CAPRICE 2; FAD2, Δ(12)-FATTY-ACID DESATURASE; FT, FLOWERING LOCUS T; GBSS, GRANULE BOUND STARCH SYNTHASE 1; GFP, GREEN FLUORESCENT PROTEIN; LAZY1, shoot gravitropism gene; LFY, LEAFY; miP1a, microProtein 1a; miP1b, microProtein 1b; NFR1α, NODULATION FACTOR KINASE 1α; P450 CYP51G1, putative cytochrome P450 CYP51G1; PDS, PHYTOENE DESATURASE; PGDH1, PHOSPHOGLYCERATE DEHYDROGENASE 1; PPV, Plum pox virus; PSAT1, PHOSPHOSERINE AMINOTRANSFERASE 1; PSTVd, Potato spindle tuber viroid; ROC5, RICE OUTERMOST CELL-SPECIFIC 5; RPP5, RECOGNITION OF PERONOSPORA PARASITICA 5; SUL, SULFUR; ToLCGV, Tomato leaf curl Gujarat virus; ToLCNDV, Tomato leaf curl New Delhi virus; TRY, TRIPTYCHON; TSWV, Tomato spotted wilt virus; TuMV, Turnip mosaic virus*.

MIGS was named (de Felippes et al., 2012) a few years later than was first reported (Montgomery et al., 2008b; Felippes and Weigel, 2009). Initial MIGS transgenes included one or more fragments from one or more target genes fused downstream of miR173 target site (Figure 1B) (de Felippes et al., 2012). miR173, as other 22 nt miRNAs, possesses the ability of triggering the production of phasiRNAs from target transcripts (Chen et al., 2010; Cuperus et al., 2010). In MIGS, miR173/AGO1-guided cleavage of the MIGS primary transcript triggers RDR6-dependent synthesis of dsRNA which is subsequently processed by DCL4 to release phased tasiRNAs that lead to the efficient silencing of target genes (Figure 1B). Interestingly, MIGS can also be triggered by other 22-nt miRNAs such as miR1514a.2 (Jacobs et al., 2016), or by miR390 (Felippes and Weigel, 2009; Singh et al., 2015), a 21-nt miRNA with unique properties for triggering tasiRNA formation from *TAS3* transcripts (Montgomery et al., 2008a) (Table 1). Because miR173 is present only in Arabidopsis and closely-related species, miR173 co-expression with MIGS transgenes is necessary to trigger tasiRNA biogenesis in non-Arabidopsis species as reported in *Medicago truncatula* (Imin et al., 2013), Petunia (Han et al., 2015), soybean (Jacobs et al., 2016), and rice (Zheng et al., 2018). Despite having been widely used in gene function studies and also to confer antiviral resistance (Table 1), the MIGS approach presents a significant risk of off-target effects due to (i) the large number of tasiRNAs generated from the MIGS construct (similarly to those from classic RNAi constructs), (ii) the generation of out-of-phase siRNAs from MIGS constructs that can accumulate to high levels as observed in Petunia (Han et al., 2015), and (iii) the possibility that MIGS-derived tasiRNAs induce transitivity as reported (Han et al., 2015). Finally, loading of MIGS-derived tasiRNA into particular AGOs cannot be controlled, and for instance only a subset of these tasiRNAs, typically those with a 5' U, will be loaded into AGO1 while others may be loaded into different AGOs or degraded.

## Appropriate Terminology to Refer to Secondary siRNA-based Silencing Tools

After having reviewed the literature, it seems necessary to make some brief remarks to improve the proper and consistent use of the terminology related to these secondary siRNA-based tools. For example, the production of tasiRNAs from a transgene including a gene fragment fused downstream to a miRNA trigger target site should always be referred to as MIGS, and not to as atasiRNA/syn-tasiRNA as observed in several works (Singh et al., 2015; Zhao et al., 2015; Guo et al., 2016), even if the MIGS cassette is inserted into a *TAS* precursor (Zhao et al., 2015; Guo et al., 2016). Also, secondary siRNAs generated from MIGS transgenes should be referred as phasiRNAs or, even better, as tasiRNAs as they are expected to act *in trans* to target the desired target gene(s), but not as atasiRNAs or syn-tasiRNAs as reported (Singh et al., 2015; Zhao et al., 2015). I suggest to use the term “artificial” or “synthetic” when referring to those transgene-derived tasiRNAs that are incorporated into precursors (*TAS* or others) as individual 21-nt guide sequences that may have been designed computationally to be highly specific in silencing the corresponding target transcript(s), to be preferentially and selectively loaded by AGO1 or to avoid transitivity effects.

## Conclusions and Future Perspectives

Still in the genome editing era dominated by CRISPR/CAS9-based technologies, we anticipate that secondary sRNA-based silencing tools will continue to be broadly used because of their unique features in allowing (i) highly specific silencing (e.g., atasiRNAs/syn-tasiRNAs), (ii) the study of genes whose complete knock-out induces lethality, (iii) multitargeting, as well as the targeting of duplicated genes, antisense transcripts and individual isoforms, and (iv) the spatio-temporal control of silencing when transgene expression is regulated with tissue specific or inducible promoters. Moreover, as gene knock-down tools, it might be possible to develop secondary siRNA-based strategies for the fine-tuning regulation of secondary siRNA activity to induce the desired degree of target gene silencing.

The atasiRNA/syn-tasiRNA approach seems especially attractive due to its multiplexing capability and high specificity, as well as for the availability of high-throughput cloning strategies and automated design tools for the simple generation of atasiRNA/syn-tasiRNA constructs (Carbonell et al., 2014; Carbonell, 2019). In particular, antiviral atasiRNAs/syn-tasiRNAs designed to target multiple sites in viral RNAs should induce a more effective and durable resistance compared to single target site targeting approaches such as amiRNAs, as the possibility that the virus mutates all target sites to break the resistance seems highly improbable. Still, a deeper knowledge of the basic mechanisms governing secondary siRNA biogenesis, mode of action, and targeting efficacy is needed to further refine these secondary siRNA-based silencing tools in view of accelerating studies of gene function and crop improvement.

## Author Contributions

The author confirms being the sole contributor of this work and has approved it for publication.

### Conflict of Interest Statement

The author declares that the research was conducted in the absence of any commercial or financial relationships that could be construed as a potential conflict of interest.
